# Supplementary material for: Single-cell measurement of plasmid copy number and promoter activity
Source: Nat Commun. 2021 Mar 5;12:1475. doi: 10.1038/s41467-021-21734-y (PMC7935883; doi:10.1038/s41467-021-21734-y)
Supplement: Supplementary file 1 — Supplementary Information [file 41467_2021_21734_MOESM1_ESM.pdf]

Supplementary Information for:

Single-cell measurement of plasmid copy number and promoter activity

Bin Shao, Jayan Rammohan, Daniel A. Anderson, Nina Alperovich, David Ross, Christopher A. Voigt

Supplementary Notes

Supplementary Note 1: Mathematical model of plasmid distribution 2

Supplementary Figures

Supplementary Figure 1: Image processing pipeline 3
Supplementary Figure 2: Imaging plasmids containing different origins of replication 4
Supplementary Figure 3: Impact of various genetic changes on the measurement of plasmid copy number 6
Supplementary Figure 4: Simulations of plasmid distributions that result from replication and partitioning 7
Supplementary Figure 5: Effect of PP7-CFP expression on transcript quantification 8
Supplementary Figure 6: Quantification of transcript copy number 9
Supplementary Figure 7: Specificity of mRNA and DNA labeling 11
Supplementary Figure 8: Imaging of labeled sgRNA 12
Supplementary Figure 9: FISH detection of mRNA after rifampicin addition 13
Supplementary Figure 10: Protein expression and plasmid copy number after rifampicin addition 14
Supplementary Figure 11: Effects of genetic modifications on DNA, mRNA and protein level 15
Supplementary Figure 12: Time-lapse measurement of DNA, mRNA and protein level 16
Supplementary Figure 13: Transcript distribution for cells with different number of plasmids 18
Supplementary Figure 14: Quantification of plasmid, mRNA and protein in different growth conditions 19
Supplementary Figure 15: mRNA and DNA labeling in different cell strains 21
Supplementary Figure 16: An example of gated flow cytometry data 22
Supplementary Figure 17: Calculation of RNAP usage for a genetic circuit 23
Supplementary Figure 18: Plasmids used in this study 24

Supplementary Tables

Supplementary Table 1: Simulation fit parameters for plasmid partitioning (a) and replication feedback (K) 26
Supplementary Table 2: List of plasmids used in this work 27
Supplementary Table 3: Genetic parts list 28
Supplementary Table 4: FISH probes used in this work 30

Reference 30

<u>Supplementary Note 1:</u> **Mathematical model of plasmid distribution**

A mechanistic model was developed to simulate the single cell plasmid distributions. The simulation consists of the following steps:

1. A population of cells (10,000) is initiated and a discrete plasmid number is assigned to each cell according to a starting distribution with mean N_0. This distribution could either be that which is experimentally derived or an arbitrary one to evaluate convergence on a solution from different initial states.
2. Remove (kill) all cells without plasmids. Of those that remain, randomly select cells and remove them until this subpopulation reduces is reduced half.
3. Cell division is simulated by randomly selecting a surviving cell to divide. If a cell has divided in this iteration already, it or its daughter will not be chosen a second time. During division, plasmid partitioning is modeled by a modified binomial process that accounts for different degrees of partitioning errors. In each round of simulation, one plasmid from the parent cell is assigned to the first daughter cell with probability:

$$p = \frac{e^{-ax_1}}{e^{-ax_1} + e^{-a(n-x_1)}} \quad . \tag{1}$$

   where x_1 is the plasmid copy number in the first daughter cell and $n - x_1$ is the plasmid copy number in the second daughter cell. n is the total number of plasmids that have been partitioned at this simulation step. a is the partitioning coefficient. If $a = 0$, each plasmid is independently assigned to each daughter with a probability of 0.5. If a is positive, plasmids are partitioned more evenly than simple binomial prediction and if a is negative, plasmids are partitioned less evenly. For plasmids without an active partitioning system (p15A, ColE1 and pUC), a is constrained to be no larger than 0. The simulation continues until all the plasmids of the parent cell are partitioned.
4. Continue Step 3 until the population returns to 10,000.
5. Simulation of plasmid replication. An exponential control curve with parameter K is used to simulate the probability of plasmid replication[1]. A plasmid is randomly selected from the full set of plasmids across the cell population and the number of plasmids in the cell containing that plasmid is recorded as N. The plasmid is then replicated with probability

$$P(N) = \frac{exp\left(-\frac{N}{K}\right)}{K} \tag{2}$$

6. Step 5 is repeated until the average plasmid copy number equals the initial value: $\langle N \rangle = N_0$.
7. Steps 1-6 are repeated until the distribution converges (Supplementary Figure 4).

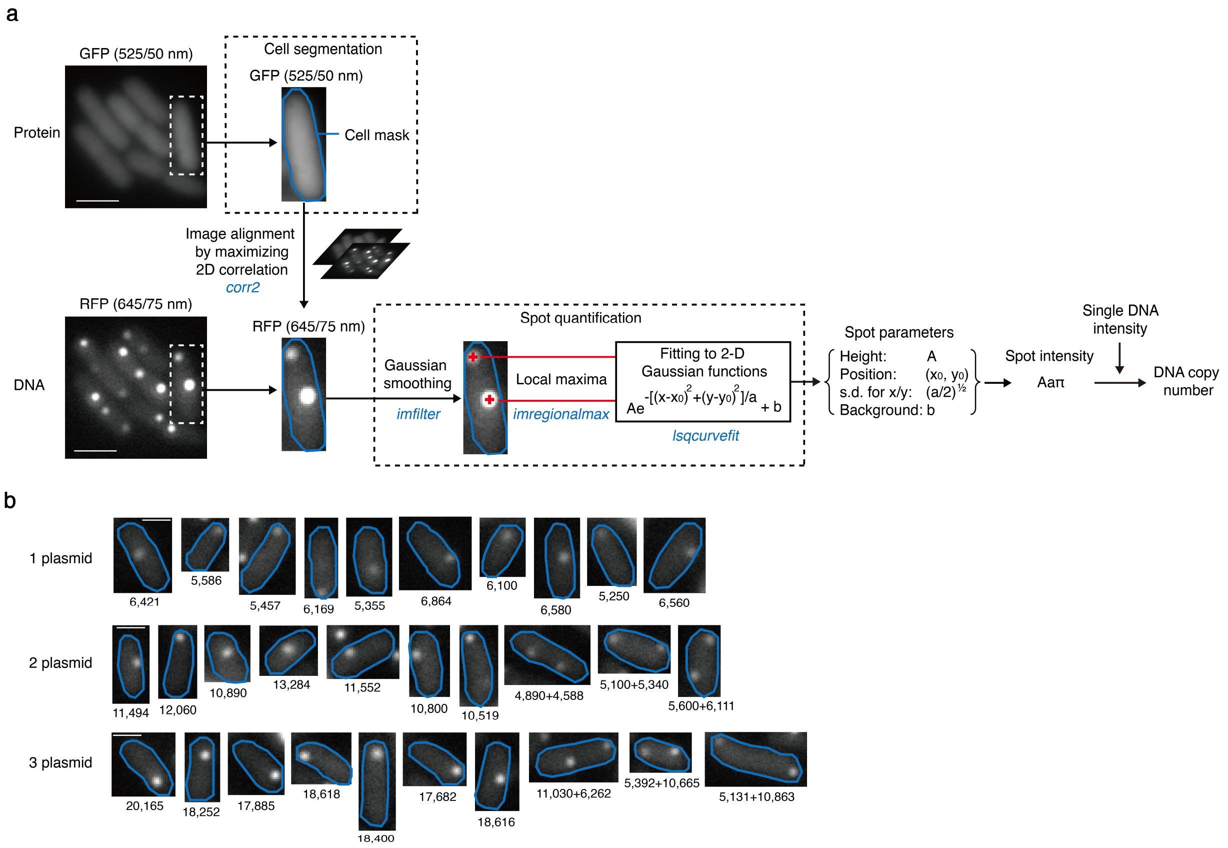

<u>Supplementary Figure 1:</u> **Image processing pipeline. (a)** Images of different color channels (525/50 nm for GFP, and 645/75 nm for DNA) were generated using microscopy (Methods). Images from GFP channel (525/50 nm) were processed using Schnitzcells[2] for cell segmentation. After image alignment, cells in the DNA (RFP) channel were identified and the RFP spot intensities were quantified as previously described (Spatzcells)[3]. The spot intensities are further converted to plasmid numbers based on the intensity of a single plasmid DNA. MATLAB functions used in each step are shown (cyan). Scale bar, 2 µm. **(b)** Sample images for cells with different numbers of plasmids. The spot intensities are shown under the images. Scale bar, 1 µm. The microscope experiments were repeated three times with similar results.

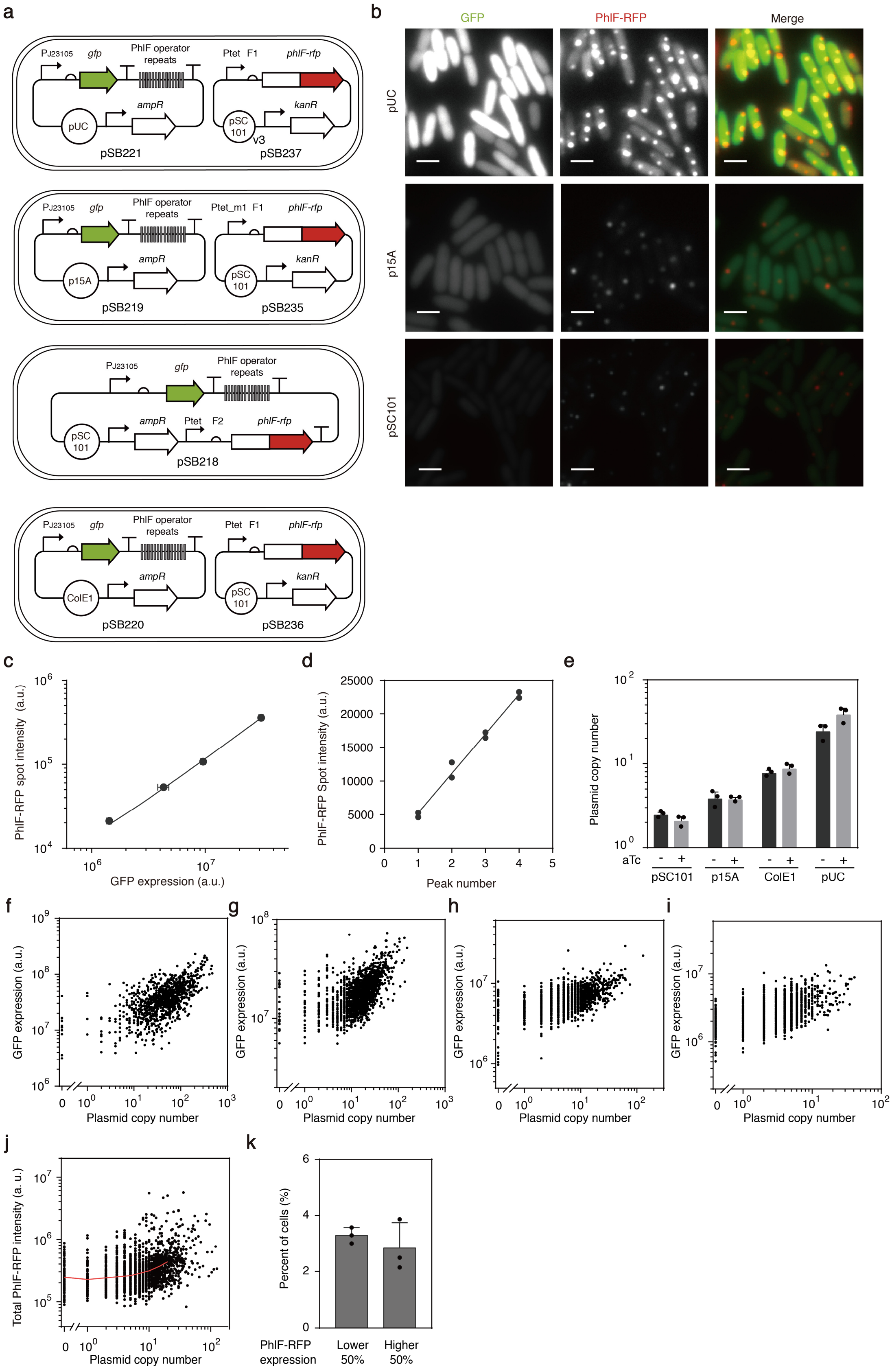

a
PJ23105
gfp
PhlF operator repeats
Ptet F1
phlF-rfp
ampR
kanR
pUC
pSC 101
v3
pSB221
pSB237
Ptet_m1 F1
p15A
pSB219
pSB235
Ptet F2
pSB218
ColE1
pSB220
pSB236
b
GFP
PhlF-RFP
Merge
pUC
p15A
pSC101
c
PhlF-RFP spot intensity (a.u.)
GFP expression (a.u.)
d
PhlF-RFP Spot intensity (a.u.)
Peak number
e
Plasmid copy number
aTc
pSC101
p15A
ColE1
pUC
f
g
h
i
GFP expression (a.u.)
Plasmid copy number
j
Total PhlF-RFP intensity (a. u.)
k
Percent of cells (%)
PhlF-RFP expression
Lower 50%
Higher 50%

Supplementary Figure 2: **Imaging plasmids containing different origins of replication. (a)** The plasmid carrying the labeling cassette (*phlF-rfp*) is shown with the plasmids being counted. Note that for the pSC101 origin, both are on the same plasmid. The sequences for genetic parts and plasmids are provided in Supplementary Tables 2 and 3. P_{J23105} is a constitutive promoter. pSC101v3 is a high copy number mutant of pSC101[4]. **(b)** Three plasmid backbones harboring *gfp* expression cassette were labeled with the PhlF-RFP fusion protein. Images for the ColE1 backbone are shown in Figure 1b. PhlF-RFP is induced with 6 ng/ml aTc (Methods). Scale bar, 2 µm. The microscope experiment was repeated three times with similar results. **(c)** Protein expression is measured by GFP fluorescence and DNA abundance measured with PhlF-RFP spot intensity. Data for four plasmids with different replication origins are shown. The black line is a linear fit that is also constrained to pass through the origin. **(d)** The spot intensity of PhlF-RFP for each peak of the histogram shown in Figure 1c. Data points are shown for two replicates performed on different days. The black line is a linear fit to the data, the slope of which provides the conversion from spot intensity to plasmid number. **(e)** qPCR was used to measure the mean plasmid copy number of plasmids containing different origins of replication. The impact of expressing PhlF-RFP from an aTc-inducible promoter (P_{tet}) is shown (6 ng/mL aTc versus no induction). Data are presented as mean values which are calculated from three experiments performed on different days and the error bars are the standard deviations. **(f)** Plasmid copy number and GFP intensity per cell for the pUC backbone (n = 1,065 cells). Each point shows one cell. **(g)** Plasmid copy number and GFP intensity per cell for the ColE1 backbone (n = 1,809 cells). **(h)** Plasmid copy number and GFP intensity per cell for the p15A backbone (n = 1,659 cells). **(i)** Plasmid copy number and GFP intensity per cell for the pSC101 backbone (n = 2,574 cells). **(j)** Plasmid copy number and total PhlF-RFP brightness per cell for the p15A backbone (n = 1,659 cells). Each point shows one cell. The red line is the average PhlF-RFP intensity for binned single cell data. **(k)** Percent of cells without detectable plasmid (p15A backbone) for cells with low and high PhlF-RFP intensity. The cell population is divided into halves based on the total PhlF-RFP intensity for each cell. Data are presented as mean values calculated from three experiments performed on different days and the error bars are the standard deviations. Source data are provided as a Source Data file.

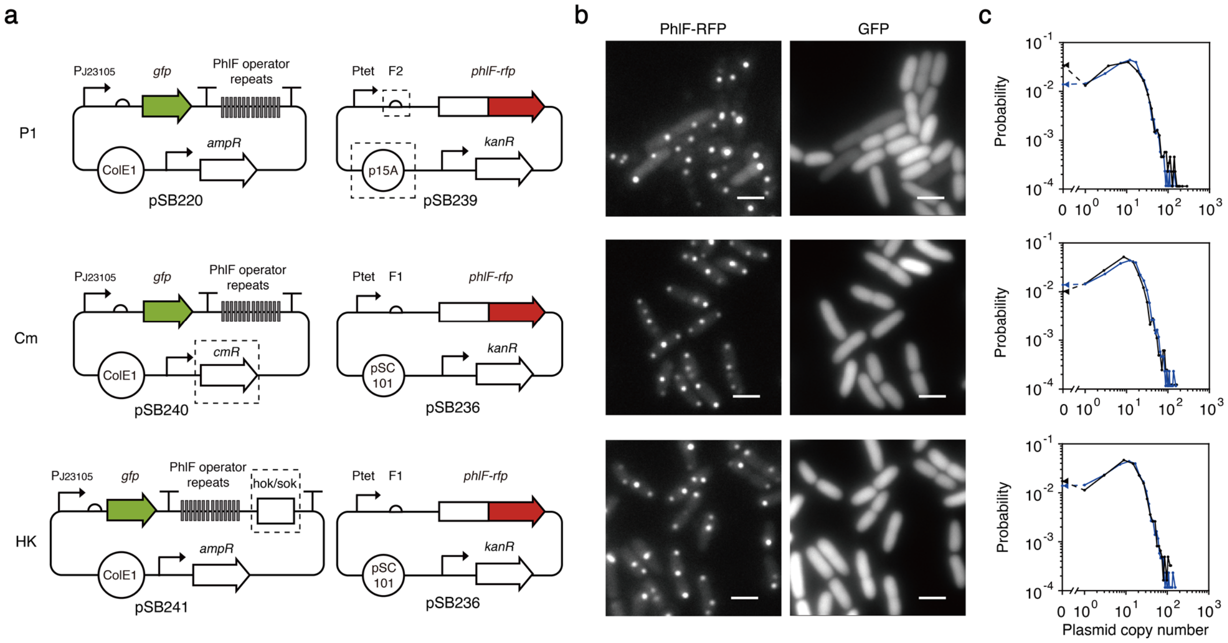

<u>Supplementary Figure 3:</u> Impact of various genetic changes on the measurement of plasmid copy number. (a) The different plasmid systems are shown, with the dashed box indicating the differences from the canonical plasmids used in this manuscript. P1: The PhlF-RFP plasmid ori is changed from pSC101 to p15A. RBS of PhlF-RFP fusion protein is changed from F1 to F2 to maintain a similar expression level. Cm: The antibiotic used is changed from kanamycin to chloramphenicol. HK: A suicide system is added to the plasmid backbone. **(b)** Images are shown for each pair of modified plasmids. Scale bar, 2 µm. The microscope experiments were repeated three times with similar results. **(c)** Copy number distributions are shown for the pairs of modified plasmids (black) and compared to the plasmids used in Figure 1b (blue). The distributions are made from a combination of three replicates performed on the same day. The numbers of cells used to generate the distributions were: 1,212 for P1, 1,425 for Cm and 1,452 for HK. P values from the two-sided Kolmogorov-Smirnov test for pooling the replicates are 0.13/0.86/0.04 for P1, 0.37/0.17/0.46 for Cm and 0.99/0.55/0.38 for HK. Dots show experimental data with lines to guide the eye. The triangles indicate the percent of cells where no plasmid is detected. Source data are provided as a Source Data file.

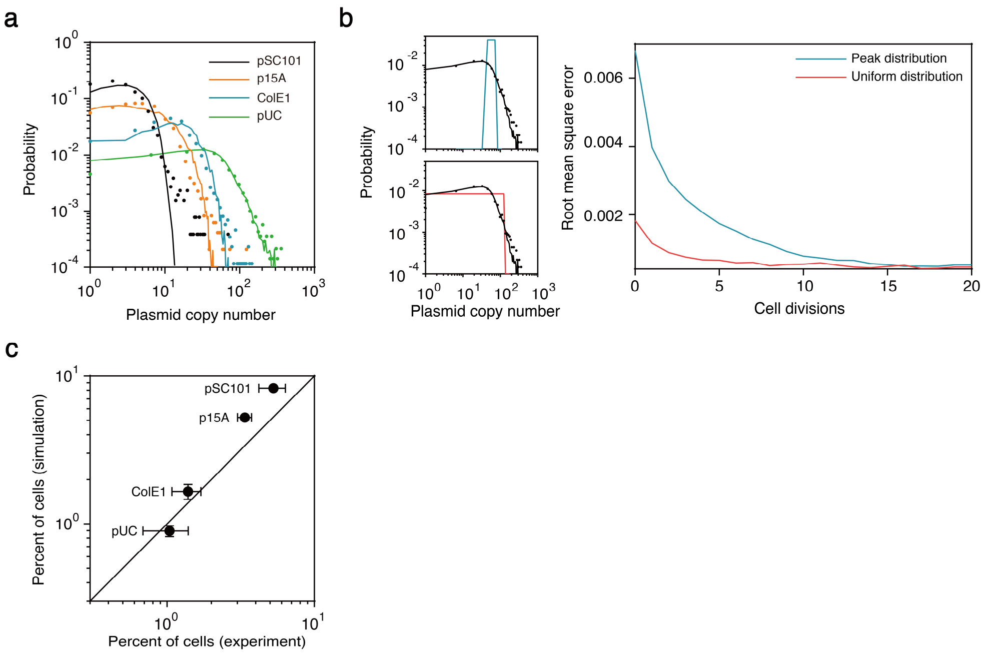

Supplementary Figure 4: **Simulations of plasmid distributions that result from replication and partitioning.** The simulation details are provided in Supplementary Note 1. **(a)** The copy number distributions for plasmids of different origins are shown (points are experimental data). The curves are fits to the model, where *a* and *K* were sampled from [-4, 4] and [0, 4000], respectively. Simulations were run three times for each combination of parameters until they converged on a plasmid distribution. The mean square error to experimental distribution is averaged from the three simulations and the parameter combination that produced the best fit is selected. The corresponding values of *K* and *a* for each plasmid origin are provided in Supplementary Table 1. The experimental data represent the combination of three replicates performed on different days. **(b)** The convergence of the simulation onto copy number distributions from different arbitrary starting distributions are shown. Each "division" represents an iteration of cell death, cell division, plasmid partitioning and plasmid replication. The final distributions onto which the simulations converge is shown in black. The data points are the experimentally-measured distribution for the pUC plasmid. **(c)** The percentage of cells without plasmids for different replication origins (bottom to top: pUC, ColE1, p15A, pSC101). Data are presented as mean values which were calculated from three experimental replicates and ten simulation replicates and the error bars represent the standard deviation. Source data are provided as a Source Data file.

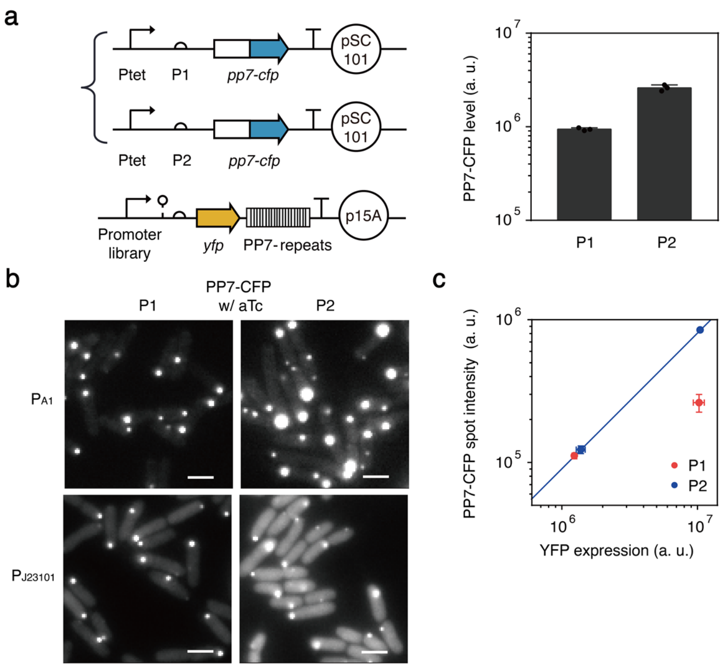

<u>Supplementary Figure 5:</u> **Effect of PP7-CFP expression on transcript quantification. (a)** Two constructs with different RBSs controlling PP7-CFP expression were compared. The P2 RBS is approximately 3-fold stronger than the P1 RBS. Data are presented as mean values of three experiments performed on the same day and the error bars are the standard deviations. Genetic part sequences are provided in Supplementary Table 3. **(b)** mRNA produced from two promoters (P_{J23101} and P_{A1}) were tagged by PP7-CFP using constructs where it is expressed using P1 or P2. PP7-CFP expression is induced with 6 ng/mL aTc. Scale bar, 2 μm. The microscope experiments were repeated three times with similar results. **(c)** YFP expression and PP7-CFP spot intensity for the P1 (red) and P2 (blue) RBSs. The blue line shows the linear fit of the data for RBS P2 (including P_{J23101} and P_{A1}), constrained to the origin. The means were calculated from three replicates performed on the same day and the error bars represent the standard deviation of these means. Source data are provided as a Source Data file.

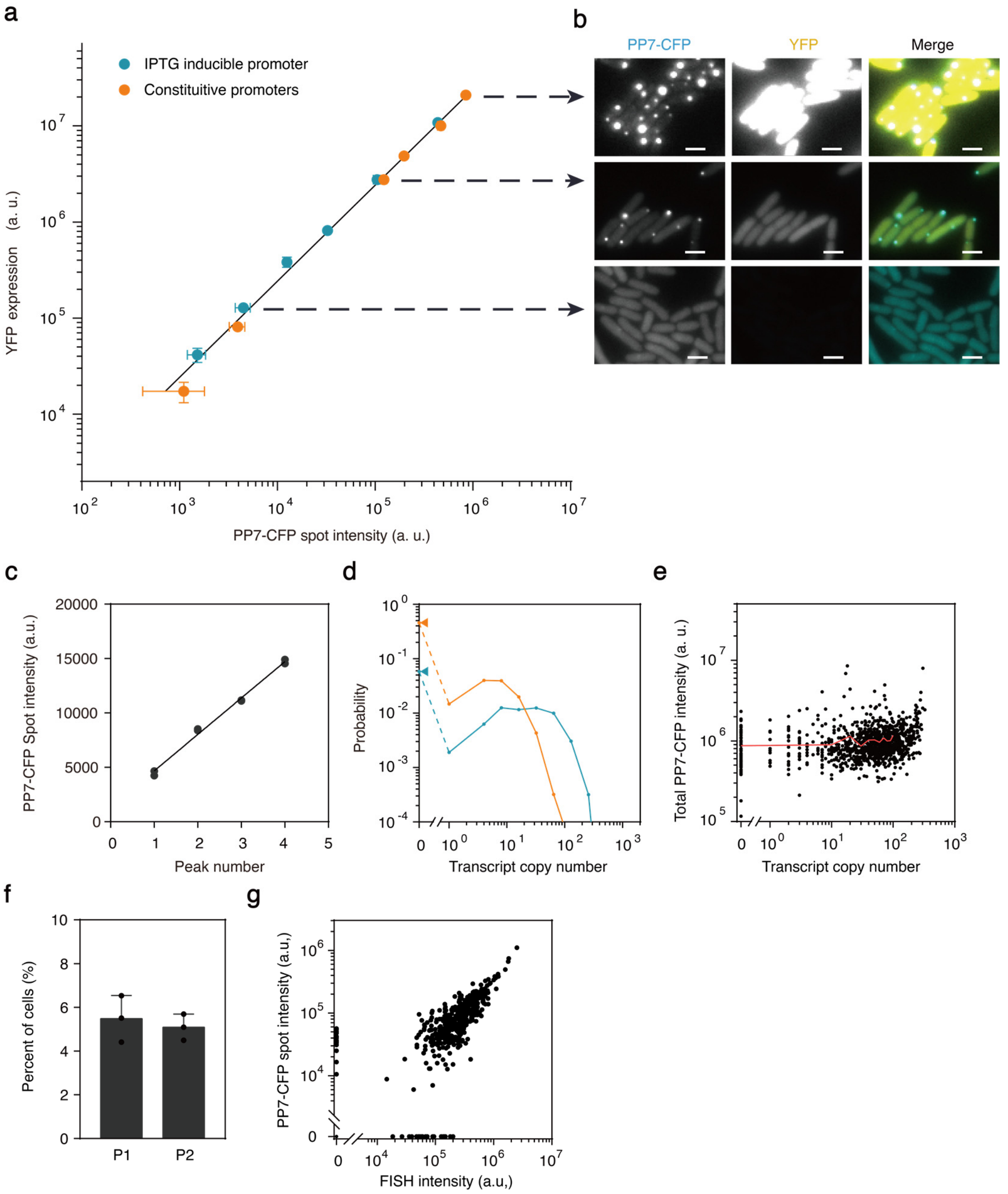

<u>Supplementary Figure 6:</u> **Quantification of transcript copy number. (a)** YFP fluorescence as a function of PP7-CFP spot intensity for a promoter library. The promoter library includes an IPTG-inducible promoter P_{tac} that is shown for six IPTG concentrations: 0, 10 µM, 20 µM, 40 µM, 100 µM, 1000 µM (cyan) and six constitutive promoters (orange) (Supplementary Table 3). In all cases, 6 ng/ml aTc was used to induce the expression of PP7-CFP. The black line shows a linear fit of the data that is constrained to pass through (0, 0). The data points are the population mean and the error bars are the standard deviation of the means

measured in three replicates performed on different days. **(b)** Representative images showing the protein expression (YFP) and transcript labeling (PP7-CFP) for different promoters (Methods). Top to bottom: P_{A1}, P_{J23101}, and P_{tac} with 20 µM IPTG. Scale bar, 2 µm. Note that for very strong promoter (P_{A1}), the YFP signal is very high, which may lead to quantification error of YFP fluoresence for neigbouring cells. This will not affect the calculation of the population mean YFP intensity in panel a. The microscope experiments were repeated three times with similar results. **(c)** The intensity of each peak is shown for the PP7-CFP spot intensity histogram in Figure 1i. The data are for a promoter that produces a small number of transcripts (P_{tac} with 20 µM IPTG). The data points represent two replicates performed on different days. The black line shows a linear fit to the data and the slope is used to convert the spot intensity to transcript copy number. **(d)** Single cell transcript copy number distribution for P_{tac} induced with different concentrations of IPTG: 20 µM (orange, n = 2,250 cells, p values from the two-sided Kolmogorov-Smirnov test for pooling the replicates are 0.18/0.34/0.97), 100 µM (cyan, n = 1,035 cells, p values: 0.23/0.04/0.89). Dots are experimental data with lines for eye guide. The triangles indicate the percent of cells where no promoter activity is detected. **(e)** Transcript copy number and total PP7-CFP intensity per cell for P_{J23101} (n = 1,126 cells). Each point shows one cell. Red line denotes the mean PP7-CFP intensity for binned single cell data. **(f)** Percent of cells without detectable transcript (P_{J23101}) for cells with low (P1) and high (P2) PP7-CFP intensity. Data are presented as mean values of three experiments performed on different days and the error bars are the standard deviations. **(g)** FISH intensity and PP7-CFP spot intensity per cell for P_{J23101} (R^2=0.95, n = 685 cells). Each spot shows one cell. Source data are provided as a Source Data file.

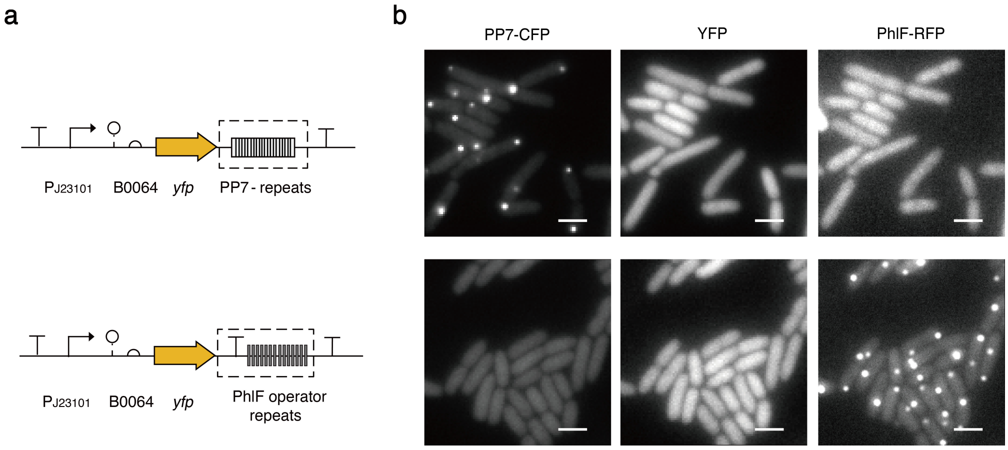

<u>Supplementary Figure 7:</u> **Specificity of mRNA and DNA labeling. (a)** Two constructs with only PP7 binding sites (pSB207) or PhlF binding sites (pSB232) are shown. Sequences for genetic parts are provided in Supplementary Table 3. **(b)** Sample images of the two constructs from a co-transformation with pSB233, which expresses the DNA- and RNA- binding fusion proteins (Methods). Scale bar, 2 µm. The microscope experiments were repeated three times with similar results.

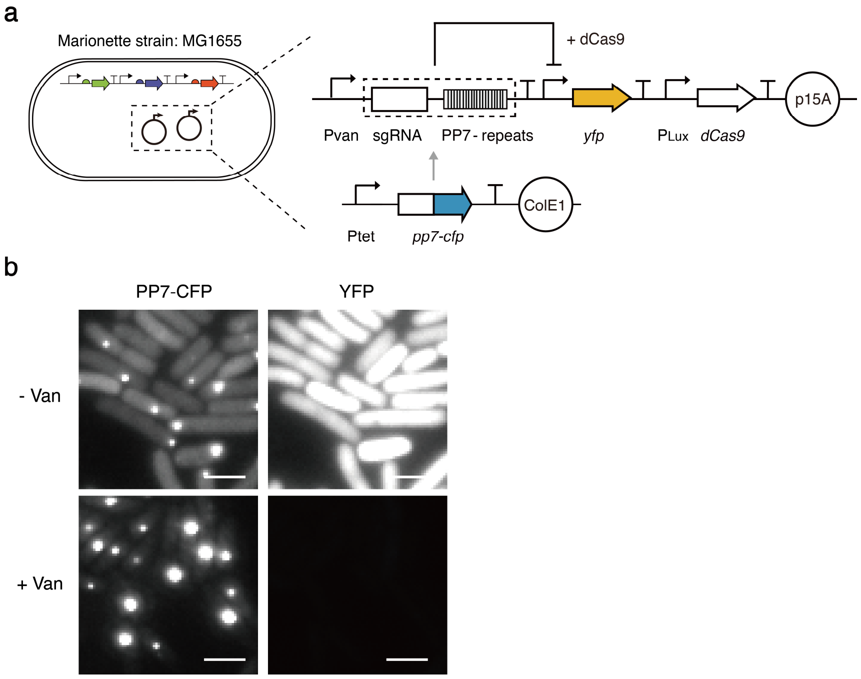

<u>Supplementary Figure 8:</u> **Imaging of labeled sgRNA.** Deactivated Cas9 (dCas9) binds to a small guide RNA (sgRNA), which directs it to a target DNA sequence[5]. Here, we sought to label the sgRNA without impact its function with dCas9. **(a)** Design of the dCas9-gate system. PP7 binding repeats are inserted after sgRNA, which targets the promoter region of a *yfp* expression cassette. The sgRNA is induced with 100 µM vanillic acid and dCas9 is induced with 1 µM 3OC6-AHL (pSB248). The PP7-CFP fusion protein is induced with 6 ng/mL aTc (pSB250). The plasmids are carried in *E. coli* MG1655 Marionette[6], which contains the inducible systems. (**b)** Sample images showing the labelled sgRNA and YFP expression. Scale bar, 2 µm. The microscope experiments were repeated three times with similar results.

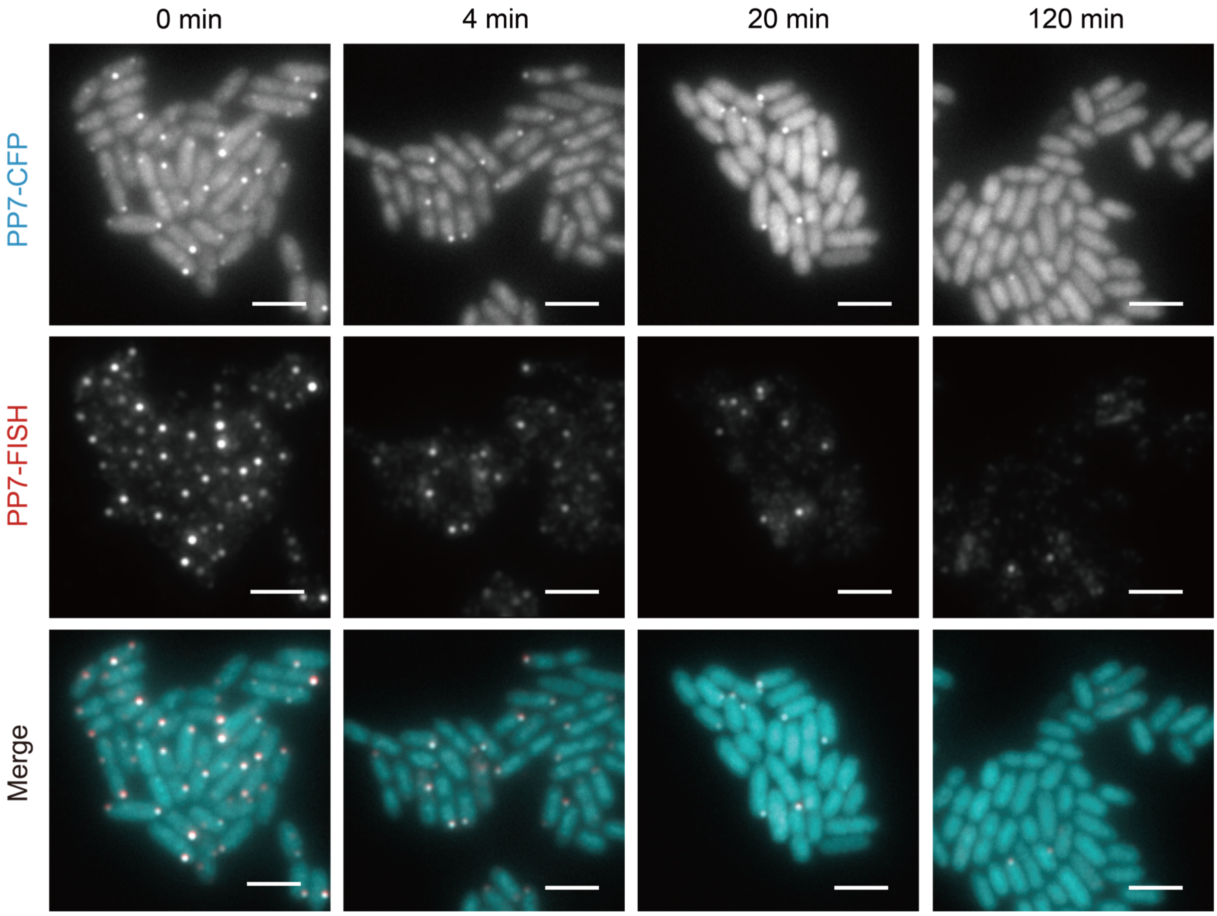

Supplementary Figure 9: **FISH detection of mRNA after rifampicin addition.** At T = 0, 500 μg/mL Rifampicin was added to stop transcription (Methods). The transcripts were expressed from P_{J23101}, carried on a p15A plasmid backbone. PP7 binding repeats on the transcripts were simultaneously detected by FISH probes (Red) and PP7-CFP fusion protein (cyan). Scale bar, 2 μm. The microscope experiments were repeated three times with similar results.

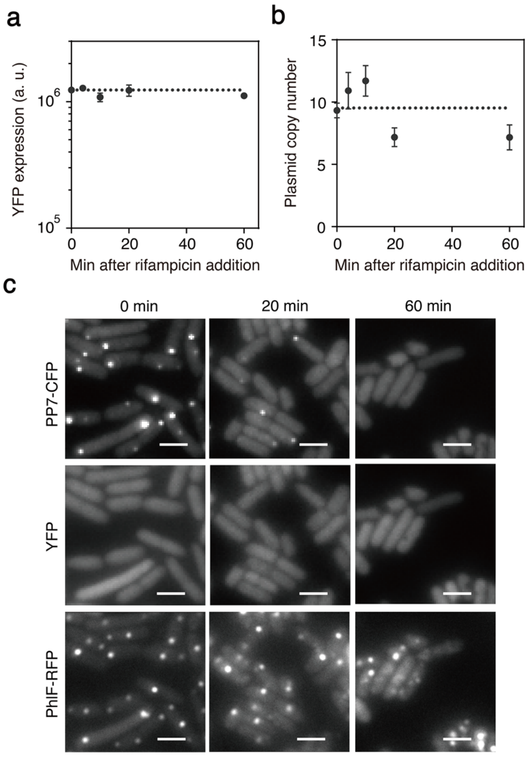

<u>**Supplementary Figure 10:**</u> **Protein expression and plasmid copy number after rifampicin addition.** **(a)** YFP expression from P_{J23101} after rifampicin addition at 0 min. The dashed line denotes the initial value. For parts a and b, the means are taken from three experiments on different days and the error bars are the standard deviation of these measurements. **(b)** The change in plasmid copy number of pSB223 (p15A ori) is shown after rifampicin addition. The dashed line denotes the initial value. **(c)** Representative images of cells after rifampicin addition. Scale bar, 2 µm. The microscope experiments were repeated three times with similar results. Source data are provided as a Source Data file.

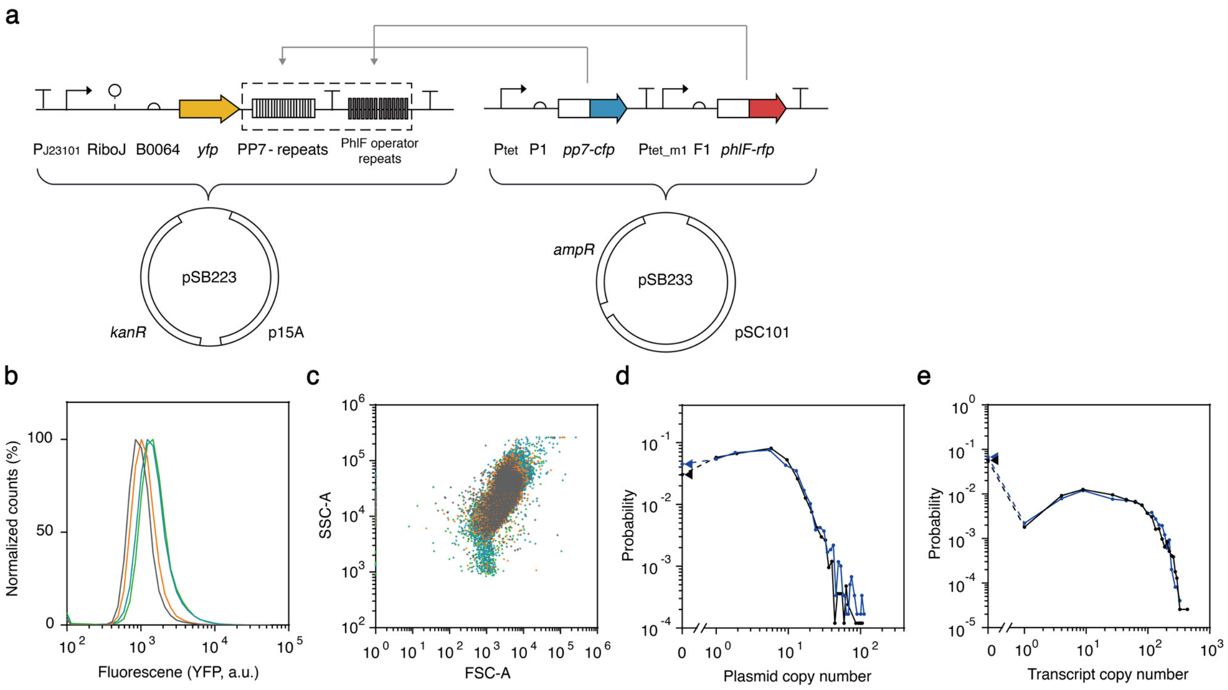

Supplementary Figure 11: **Effects of genetic modifications on DNA, mRNA and protein level. (a)** The complete two plasmid system for tripartite measurements is shown; genetic part sequences are provided in Supplementary Table 3. The plasmid maps are shown in Supplementary Figure 15. **(b)** The fluorescence disributions obtained through flow cytometry are shown. The black distribution is for cells carrying pSB201, which contains the YFP expression casette, but no operator repeats (Supplementary Figure 15). The orange distribution contains the PP7 and PhlF repeats (pSB223, part a). The cyan and green distributions show the inclusion of the pSB233 plasmid (part a) in the absence and presence of 6 ng/mL aTc, respectively. **(c)** FSC and SSC plot of cells by flow cytometry and colored as in part b. **(d)** The impact of the PP7 operator repeat region on the plasmid copy number calculation is shown. The distributions are from n = 3,999 cells analyzed by microscopy. The black distribution is for pSB223 and the blue is for this plasmid where the PP7 operator repeat region is removed (pSB232, Supplementary Figure 15). P values from the two-sample Kolmogorov-Smirnov test for pooling the replicates are 0.92/0.18/0.08. **(e)** The impact of the PhlF repeat region on the calculation of mRNA copy number. The distributions are from n = 3,564 cells analyzed by microscopy. The black distribution is for pSB223 and the blue distribution is for this plasmid where the PhlF operator region is removed (pSB207, Supplementary Figure 15). P values from the two-sample Kolmogorov-Smirnov test for pooling the replicates are 0.61/0.08/0.19. For part d and e, dots are experimental data with lines for eye guide. The triangles indicate the percent of cells where no transcript or plasmid is detected. Source data are provided as a Source Data file.

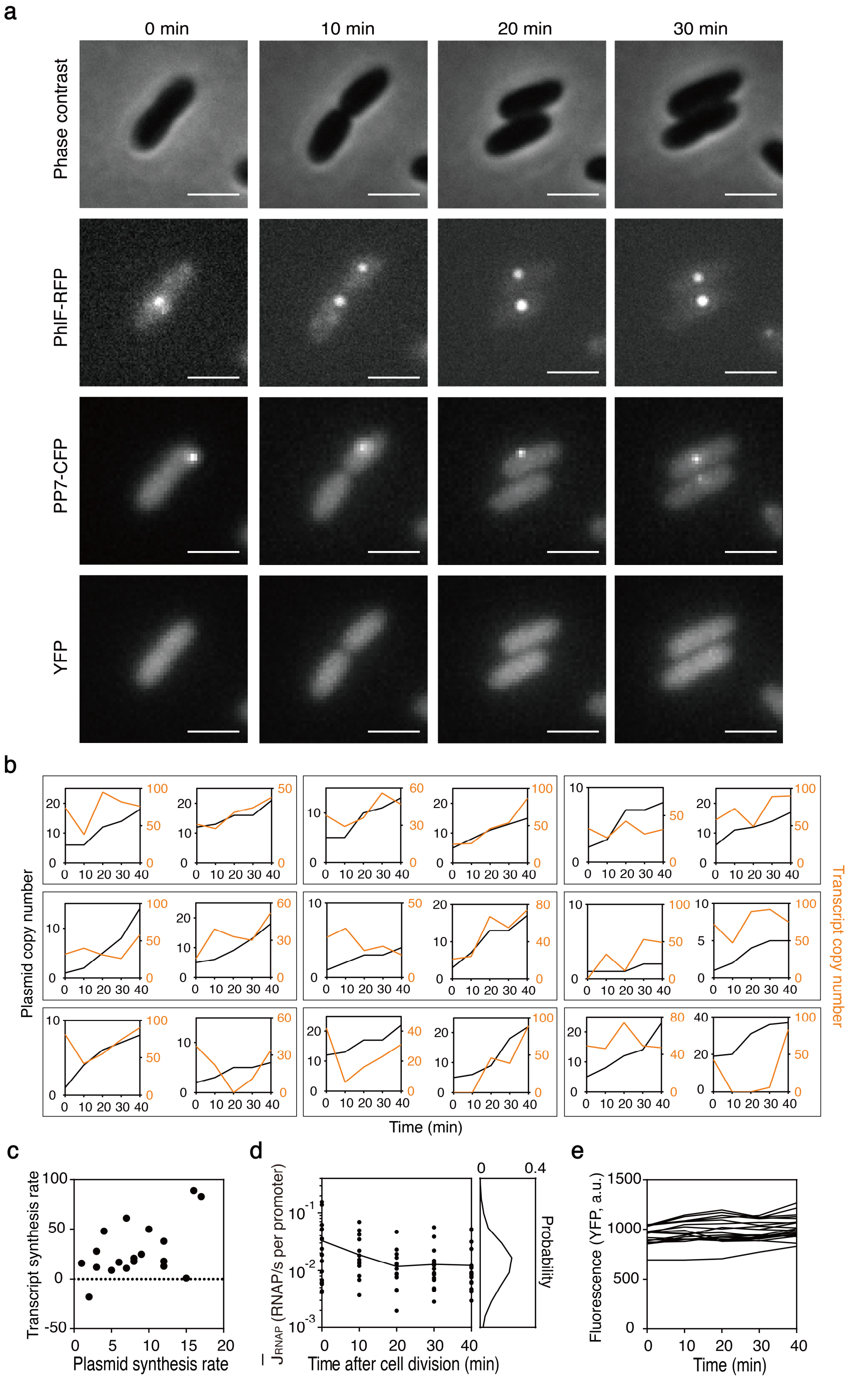

Supplementary Figure 12: **Time-lapse measurement of DNA, mRNA and protein level. (a)** Time-laspe measurement of DNA (RFP), mRNA (CFP) and protein (YFP) in *E. coli* cells (NEB10-beta). Cells were transformed with pSB223 and pSB234 and grown on agar pad with 6 ng/mL aTc for PhlF-RFP and PP7-CFP induction (Methods). Scale bar 2 µm**.** The microscope experiments were repeated three times with similar results. **(b)** Timecourse of plasmid (black) and transcript copy number (orange) for individual cells. T = 0

corresponds to cell division. Two daugher cells from the same mother are grouped by the black boxes. **(c)** Plasmid and transcript synthesis rate for individual cells. The synthesis rates are quantified as the increase of plasmid or transcript copy number from 10 min to 40 min after cell division. Each dot represents a measurement from one cell. **(d)** Promoter activity as a function of time after cell division. Each dot represents promoter activity of a single cell and the average is shown in blackline. The promoter activity distribution is shown on the right. **(e)** Timecourse of protein concentration for single cells. T = 0 corresponds to cell division. Source data are provided as a Source Data file.

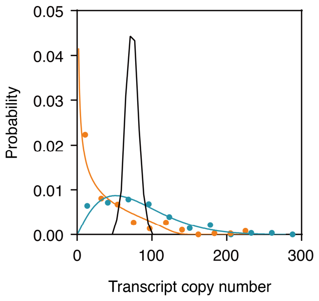

<u>Supplementary Figure 13:</u> Transcript distribution for cells with different number of plasmids. These data correspond to that shown in Figure 2a, but where the cells have been binned by plasmid copy number. The orange distribution shows the distribution of mRNA transcripts for those cells that have 2 plasmids (n = 153 cells). The cyan distribution shows the distribution for cells that have 10 plasmids (n = 104 cells). The dots are experimental measurements and solid lines are fit from a two-state promoter model[7] using the finite state projection algorithm[8]. For the latter distribution, a Possion distribution with the same mean (black) is shown for reference. Source data are provided as a Source Data file.

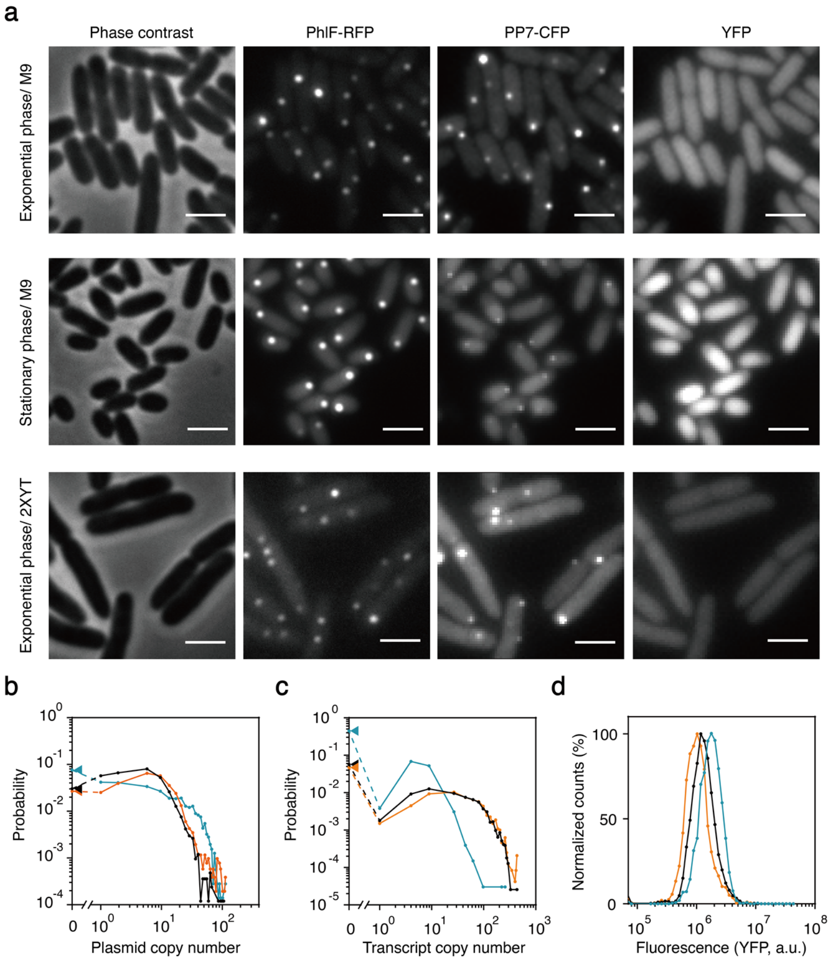

Supplementary Figure 14: **Quantification of plasmid, mRNA and protein in different growth conditions.** **(a)** Sample images showing simultaneous quantification of DNA (RFP), RNA copy number (CFP) and protein production (YFP) for P_{J23101} carried on pSB223. For cells growing in M9 media in exponential growth phase, PP7-CFP and PhlF-RFP are induced with 6 ng/mL aTc (pSB233). For mRNA and DNA counting in stationry phase, 6 ng/mL aTc is used to induce fusion protein expression from pSB233 in early stationary phase (Methods). For mRNA and DNA counting in 2×YT media, 6 ng/mL aTc is used to induce fusion protein expression from pSB234 in exponential phase (Methods). Cells in 2×YT media have larger growth rate (protein dilution rate), so the plasmid with higher PP7-CFP/PhlF-RFP expression is used (pSB234). Scale bar, 2 µm. The microscope experiments were repeated three times with similar results. **(b)** Plasmid distributions for different growth conditions. The black line shows the plasmid distribution in M9 media in exponential growth phase (p values from the two-sample Kolmogorov-Smirnov test for pooling the replicates are 0.64/0.12/0.07). The orange line shows the plasmid distributon in 2×YT media in exponential

growth phase (p values from the two-sample Kolmogorov-Smirnov test: 0.30/0.12/0.04) and the cyan line shows the plasmid distribution in M9 media in stationary phase (p values from the two-sample Kolmogorov-Smirnov test: 0.04/0.59/0.19). **(c)** Transcript distributions for different growth conditions, colored as in part b. (p values from the two-sample Kolmogorov-Smirnov test are 0.51/0.02/0.21 for M9 media in exponential growth phase, 0.75/0.04/0.20 for 2×YT media in exponential growth phase and 0.64/0.30/0.69 for M9 media in stationary phase). **(d)** The YFP fluorescence distributions for different growth conditions are shown, colored as in part b. All the distributions are made from a combination of three replicates performed on different days. For part b, c and d, dots are experimental data with lines to guide the eye. For part b and c, the triangles indicate the percent of cells where no plasmid or transcript is detected. Source data are provided as a Source Data file.

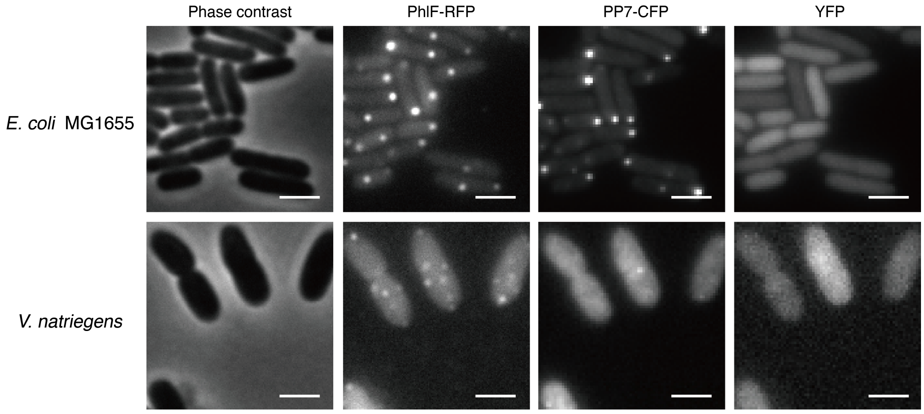

<u>Supplementary Figure 15:</u> **mRNA and DNA labeling in different cell strains.** *E. coli* MG1655 cells were transformed with pSB223 and pSB233 and grown in M9 media in exponential growth phase. *V. natriegens* cells were transformed with pSB223 and pSB234 and grown in M9 media with 2% NaCl in exponential growth phase (Methods). PP7-CFP and PhlF-RFP expressions are induced with 6 ng/mL aTc. Scale bar 2 µm. The microscope experiments were repeated three times with similar results.

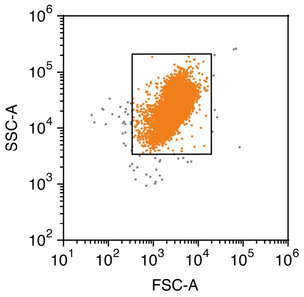

<u>Supplementary Figure 16:</u> An example of gated flow cytometry data. *E. coli* cells (orange, 49842 events) are separted from the debris (grey, 158 events) by the gating strategy: 350 < FSC-A < 20,000 and 3,500 < SSC-A < 200,000. FSC-A and SSC-A are the forward-scattered and side-scattered lights for each event, respectively. Data are shown for cells carrying pSB201, which contains the YFP expression casette. Source data are provided as a Source Data file.

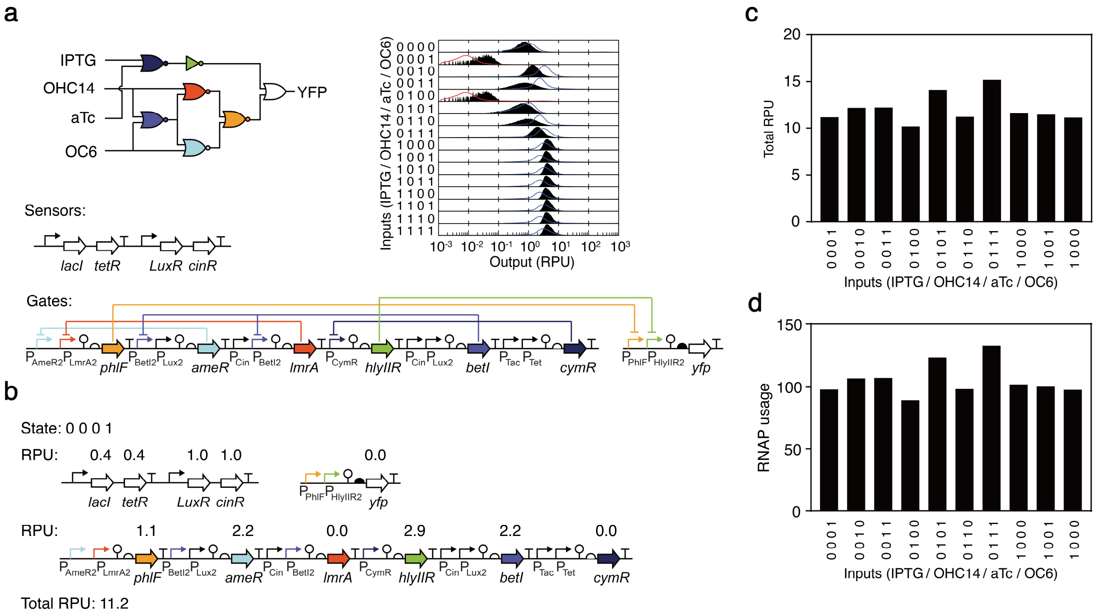

<u>Supplementary Figure 17:</u> **Calculation of RNAP usage for a genetic circuit. (a)** Genetic design for segment A in 7-segment circuits[9]. The predicted (red for off state and blue for on state) and measured (black) fluorescence outputs are shown for different input combinations. The order of the input inducer is 0.2 mM IPTG, 1 µM OHC14, 2 ng/ml aTc, 0.1 µM OC6. 0 is for absence of inducer and 1 is for presence of inducer. Data reproduced from Shin *et al*[9]. **(b)** Response of all the sensors and gates in state (0 0 0 1). **(c)** Total RPU of the circuit in each state. **(d)** RNAP usage converted from RPU values. The conversion is done as follows: the total RNAP flux is RPU × 0.02 s^{-1}. The average gene length (L) is assumed to be 1 kb, the RNAP elongation rate (v) is estimated to be ~20 nt/s[10, 11]. The gene copy number (N) is 9. Then the RNAP usage is RPU × 0.02×(LN/v). Source data are provided as a Source Data file.

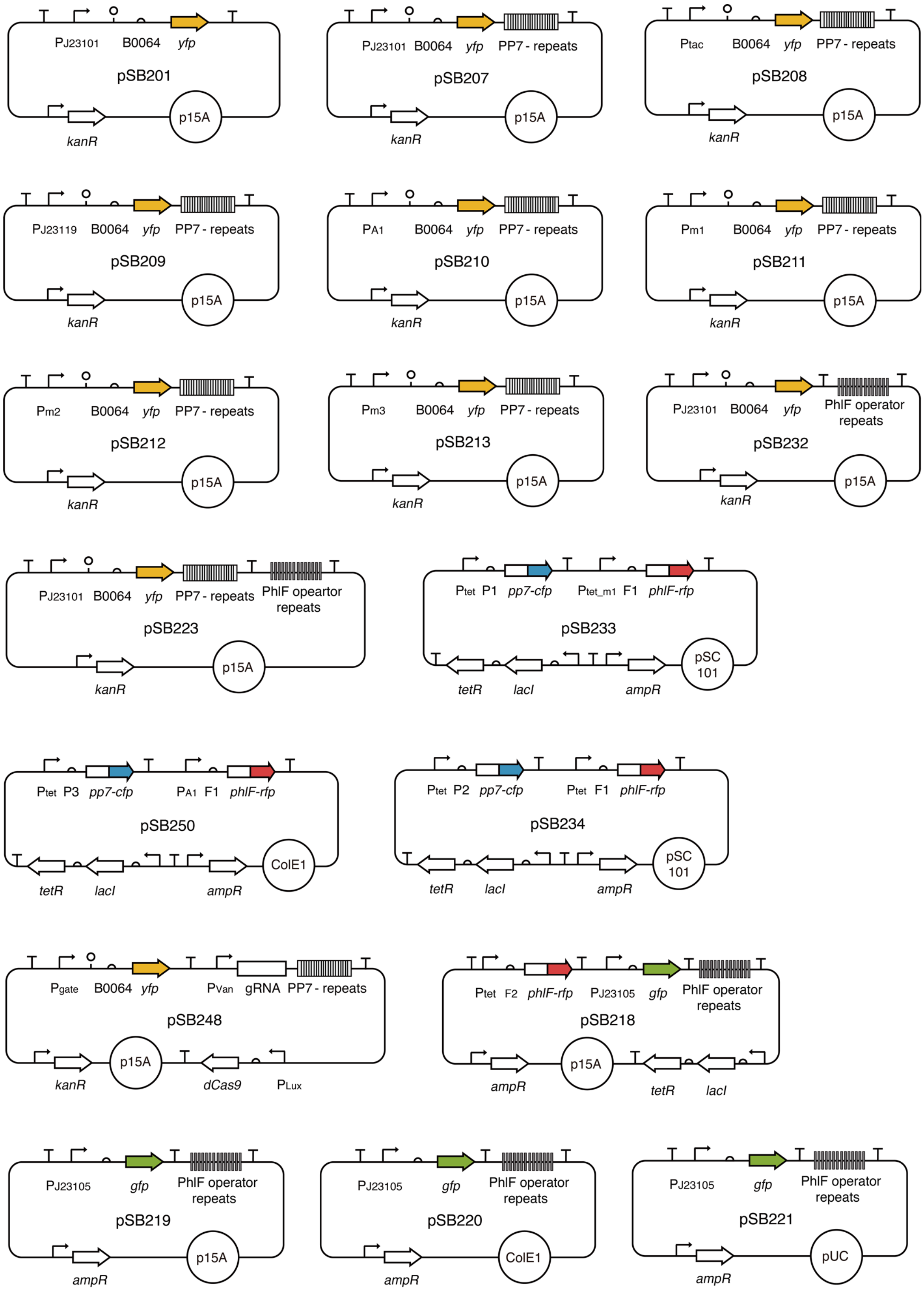

PJ23101 B0064 yfp
pSB201
kanR
p15A
PJ23101 B0064 yfp PP7 - repeats
pSB207
kanR
p15A
Ptac B0064 yfp PP7 - repeats
pSB208
kanR
p15A
PJ23119 B0064 yfp PP7 - repeats
pSB209
kanR
p15A
PA1 B0064 yfp PP7 - repeats
pSB210
kanR
p15A
Pm1 B0064 yfp PP7 - repeats
pSB211
kanR
p15A
Pm2 B0064 yfp PP7 - repeats
pSB212
kanR
p15A
Pm3 B0064 yfp PP7 - repeats
pSB213
kanR
p15A
PJ23101 B0064 yfp PhlF operator repeats
pSB232
kanR
p15A
PJ23101 B0064 yfp PP7 - repeats PhlF opeartor repeats
pSB223
kanR
p15A
Ptet P1 pp7-cfp Ptet_m1 F1 phlF-rfp
pSB233
tetR lacI ampR
pSC 101
Ptet P3 pp7-cfp PA1 F1 phlF-rfp
pSB250
tetR lacI ampR
ColE1
Ptet P2 pp7-cfp Ptet F1 phlF-rfp
pSB234
tetR lacI ampR
pSC 101
Pgate B0064 yfp PVan gRNA PP7 - repeats
pSB248
kanR p15A dCas9 PLux
Ptet F2 phlF-rfp PJ23105 gfp PhlF operator repeats
pSB218
ampR p15A tetR lacI
PJ23105 gfp PhlF operator repeats
pSB219
ampR
p15A
PJ23105 gfp PhlF operator repeats
pSB220
ampR
ColE1
PJ23105 gfp PhlF operator repeats
pSB221
ampR
pUC

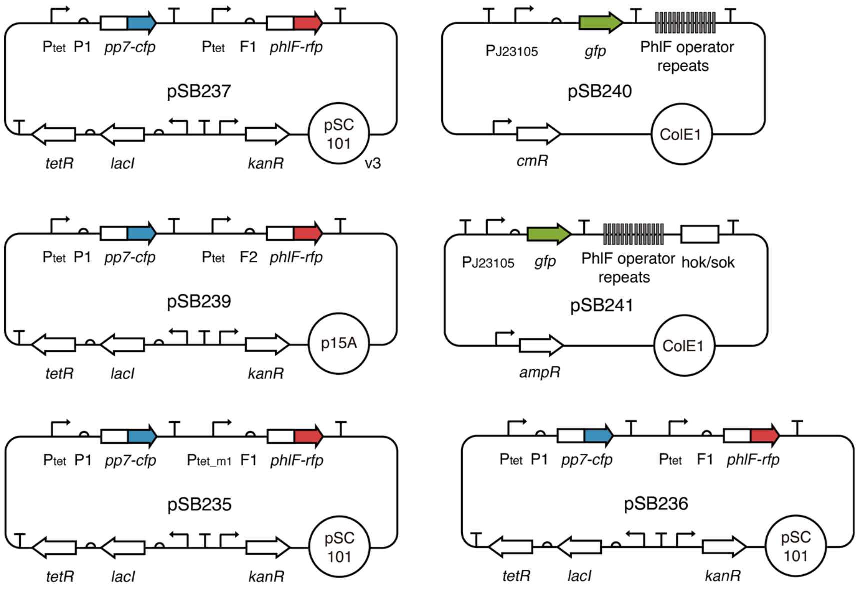

Supplementary Figure 18: Plasmids used in this study.

Table S1: Simulation fit parameters for plasmid partitioning (a) and replication feedback (K)

	Plasmid origin of replication (ori)			
Parameter	pSC101	p15A	ColE1	pUC
a	1.9 ± 0.1	$-1.5 \pm 0.1\times10^{-1}$	$-1.4 \pm 0.2\times10^{-1}$	$-5.0 \pm 0.5\times10^{-2}$
K	8 ± 1	28 ± 2	29 ± 1	$8.0 \pm 0.4\times10^{2}$
N_0	4	9	18	61

Table S2: List of plasmids used in this work

Plasmid Name	Ori	Marker	Description
pSB201	p15A	Kan	Unmodified RPU plasmid
pSB207	p15A	Kan	Transcript calibration: P_{J23101}-*yfp*-PP7X20
pSB208	p15A	Kan	Transcript calibration: P_{tac}- *yfp* -PP7X20
pSB209	p15A	Kan	Transcript calibration: P_{J23119}- *yfp* -PP7X20
pSB210	p15A	Kan	Transcript calibration: P_{A1}- *yfp* -PP7X20
pSB211	p15A	Kan	Transcript calibration: P_{m1}- *yfp* -PP7X20
pSB212	p15A	Kan	Transcript calibration: P_{m2}- *yfp* -PP7X20
pSB213	p15A	Kan	Transcript calibration: P_{m3}- *yfp* -PP7X20
pSB218	pSC101	Amp	Plasmid calibration: pSC101 with PhlFX14
pSB219	p15A	Amp	Plasmid calibration: p15A with PhlFX14
pSB220	ColE1	Amp	Plasmid calibration: ColE1 with PhlFX14
pSB221	pUC	Amp	Plasmid calibration: pUC with PhlFX14
pSB223	p15A	Kan	New measurement standard
pSB232	p15A	Kan	Modified RPU with only PhlFX14
pSB233	pSC101	Amp	P_{tet} driving *pp7-cfp* (RBS: P1) and *phlF-rfp*
pSB234	pSC101	Amp	P_{tet} driving *pp7-cfp* (RBS: P2) and *phlF-rfp*
pSB235	pSC101	Kan	P_{tet_m1} driving *phlF-rfp*
pSB236	pSC101	Kan	P_{tet} driving *phlF-rfp*
pSB237	pSC101v3	Kan	P_{tet} driving *phlF-rfp*
pSB239	p15A	Kan	P_{tet} driving *phlF-rfp*
pSB240	ColE1	Cm	ColE1 with PhlFX14
pSB241	ColE1	Amp	ColE1 with hok/sok system
pSB248	p15A	Kan	dCas9-gate, PP7 repeats in the end of stem loop 3
pSB250	ColE1	Amp	P_{tet} driving *pp7-cfp* and P_{A1} driving *phlF-rfp*

Table S3: Genetic parts list

Name	Sequence	References
**Promoters**		
P_{J23101}	GATAAGTCCCTAACTTTTACAGCTAGCTCAGTCCTAGGTATTATGCTAGC	Part:BBa_J23101(parts.igem.org)
P_{J23119}	GATAAGTCCCTAACTTTGACAGCTAGCTCAGTCCTAGGTATAATGCTAGC	Part:BBa_J23119(parts.igem.org)
P_{J23105}	TTTACGGCTAGCTCAGTCCTAGGTACTATGCTAGC	Part:BBa_J23105(parts.igem.org)
P_{A1}	GATAAGTCCCTAACTTTATCAAAAAGAGTATTGACTTAAAGTCTAACCTATAGGATACTACAGCCA	12
P_{m1}	GATAAGTCCCTAACTTTTACAGCTAGCTCAGTCCTAGGAAACGGGCTAGCCTGA	This study
P_{m2}	GATAAGTCCCTAACTTTTACAGCTAGCTCAGTCCTAGGGCGTCCGCTAGCCTGA	This study
P_{m3}	GATAAGTCCCTAACTTTTACAGCTAGCTCAGTCCTAGGTTTTGTGCTAGCCTGA	This study
P_{tac}	AACGATCGTTGGCTGTGTTGACAATTAATCATCGGCTCGTATAATGTGTGGAATTGTGAGCGCTCACAATT	13
P_{tet}	TACTCCACCGTTGGCTTTTTTCCCTATCAGTGATAGAGATTGACATCCCTATCAGTGATAGAGATAATGAGCAC	13
P_{tet_m1}	TACTCCACCGTTGGCTTTTTTCCCTATCAGTGATAGAGATTGACATCCCTATCAGTGATAGAGATACTGAGCAC	This study
P_{gate}	TTTACACCACTAGCTAGAGGGGGTATTATGCTAGC	This study
P_{Van}	ATTGGATCCAATTGACAGCTAGCTCAGTCCTAGGTACCATTGGATCCAAT	6
P_{Lux}	ACCTGTAGGATCGTACAGGTTTACGCAAGAAAATGGTTTGTTACTTTCGAATAAA	6
**RBS**		
B0064	AAAGAGGGGAAA	Part:BBa_B0064(parts.igem.org)
B0034	AAAGAGGAGAAA	Part:BBa_B0034(parts.igem.org)
P1	AGAGAAAGAGGTCAAATACTAG	This study
P2	AGAGAAAGAGGGTAAATACTAG	This study
P3	AGAGAAAGAGCAGAAATACTAG	This study
F1	GGAGCTATGGACTATGTTTGAAAGGCTGAAATACTAG	13
F2	AAAGAGGGAAAA	This study
**Terminators**		
L3S3P21	CCAATTATTGAAGGCCTCCCTAACGGGGGGCCTTTTTTTGTTTCTGGTCTCCC	14
L3S2P21	CTCGGTACCAAATTCCAGAAAAGAGGCCTCCCGAAAGGGGGGCCTTTTTTCGTTTTGGTCC	14
L3S3P11	CCAATTATTGAACACCCTTCGGGGTGTTTTTTTGTTTCTGGTCTACC	14
L3S1P13	GACGAACAATAAGGCCTCCCTAACGGGGGGCCTTTTTTATTGATAACAAAA	14
L3S2P55	CTCGGTACCAAAGACGAACAATAAGACGCTGAAAAGCGTCTTTTTTCGTTTTGGTCC	14
B0010	CCAGGCATCAAATAAAACGAAAGGCTCAGTCGAAAGACTGGGCCTTTCGTTTTATCTGTTGTTTGTCGGTGAACGCTCTC	Part:BBa_B0010(parts.igem.org)
**Ribozymes**		
RiboJ	AGCTGTCACCGGATGTGCTTTCCGGTCTGATGAGTCCGTGAGGACGAAACAGCCTCTACAAATAATTTTGTTTAA	15
**Genes**		
pp7	ATGTCCAAAACCATCGTTCTTTCGGTCGGCGAGGCTACTCGCACTCTGACTGAGATCCAGTCCACCGCAGACCGTCAGATCTTCGAAGAGAAGGTCGGGCCTCTGGTGGGTCGGCTGCGCCTCACGGCTTCGCTCCGTCAAAACGGAGCCAAGACCGCGTATCGCGTCAACCTAAAACTGGATCAGGCGGACGTCGTTGATTGCTCCACCAGCGTCTGCGGCGAGCTTCCGAAAGTGCGCTACACTCAGGTATGGTCGCACGACGTGACAATCGTTGCGAATAGCACCGAGGCCTCGCGCAAATCGTTGTACGATTTGACCAAGTCCCTCGTCGCGACCTCGCAGGTCGAAGATCTTGTCGTCAACCTTGTGCCGCTGGGCCGT	16
phlF	ATGGCACGTACCCCGAGCCGTAGCAGCATTGGTAGCCTGCGTAGTCCGCATACCCATAAAGCAATTCTGACCAGCACCATTGAAATCCTGAAAGAATGTGGTTATAGCGGTCTGAGCATTGAAAGCGTTGCACGTCGTGCCGGTGCAAGCAAACCGACCATTTATCGTTGGTGGACCAATAAAGCAGCACTGATTGCCGAAGTGTATGAAAATGAAAGCGAACAGGTGCGTAAATTTCCGGATCTGGGTAGCTTTAAAGCCGATCTGGATTTTCTGCTGCGTAATCTGTGGAAAGTTTGGCGTGAAACCATTTGTGGTGAAGCATTTCGTTGTGTTATTGCAGAAGCACAGCTGGACCCTGCAACCCTGACCCAGCTGAAAGATCAGTTTATGGAACGTCGTCGTGAGATGCCGAAAAAACTGGTTGAAAATGCCATTAGCAATGGTGAACTGCCGAAAGATACCAATCGTGAACTGCTGCTGGATATGATTTTTGGTTTTTGTTGGTATCGCCTGCTGACCGAACAGCTGACCGTTGAACAGGATATTGAAGAATTTACCTTCCTGCTGATTAATGGTGTTTGTCCGGGTACACAGCGT	13

cfp	ATGCGTAAAGGCGAAGAGCTGTTCACTGGTGTCGTCCCTATTCTGGTGGAACTGGATGGTGATGTCAACGGTCATAAGTTTTCCGTGCGTGGCG<br>AGGGTGAAGGTGACGCAACTAATGGTAAACTGACGCTGAAGTTCATCTGTACTACTGGTAAACTGCCGGTACCTTGGCCGACTCTGGTAACGAC<br>GCTGACTTGGGGTGTTCAGTGCTTTGCTCGTTATCCGGACCATATGAAGCAGCATGACTTCTTCAAGTCCGCCATGCCGGAAGGCTATGTGCAG<br>GAACGCACGATTTCCTTTAAGGATGACGGCACGTACAAAACGCGTGCGGAAGTGAAATTTGAAGGCGATACCCTGGTAAACCGCATTGAGCTGA<br>AAGGCATTGACTTTAAAGAAGACGGCAATATCCTGGGCCATAAGCTGGAATACAATTACATCAGCGACAATGTTTACATCACCGCCGATAAACA<br>AAAAAATGGCATTAAAGCGAATTTTAAAATTCGCCACAACGTGGAGGATGGCAGCGTGCAGCTGGCTGATCACTACCAGCAAAACACTCCAATC<br>GGTGATGGTCCTGTTCTGCTGCCAGACAATCACTATCTGAGCACGCAAAGCGTTCTGTCTAAAGATCCGAACGAGAAACGCGATCATATGGTTC<br>TGCTGGAGTTCGTAACCGCAGCGGGCATCACGCATGGTATGGATGAACTGTACAAATGATAA	17
rfp	ATGGTGAGCAAGGGCGAGGAGGATAACATGGCCATCATCAAGGAGTTCATGCGCTTCAAGGTGCACATGGAGGGCTCCGTGAACGGCCACGAGT<br>TCGAGATCGAGGGCGAGGGCGAGGGCCGCCCCTACGAGGGCACCCAGACCGCCAAGCTGAAGGTGACCAAGGGTGGCCCCCTGCCCTTCGCCTG<br>GGACATCCTGTCCCCTCAGTTCATGTACGGCTCCAAGGCCTACGTGAAGCACCCCGCCGACATCCCCGACTACTTGAAGCTGTCCTTCCCCGAG<br>GGCTTCAAGTGGGAGCGCGTGATGAACTTCGAGGACGGCGGCGTGGTGACCGTGACCCAGGACTCCTCCTTGCAGGACGGCGAGTTCATCTACA<br>AGGTGAAGCTGCGCGGCACCAACTTCCCCTCCGACGGCCCCGTAATGCAGAAGAAGACCATGGGCTGGGAGGCCTCCTCCGAGCGGATGTACCC<br>CGAGGACGGCGCCCTGAAGGGCGAGATCAAGCAGAGGCTGAAGCTGAAGGACGGCGGCCACTACGACGCTGAGGTCAAGACCACCTACAAGGCC<br>AAGAAGCCCGTGCAGCTGCCCGGCGCCTACAACGTCAACATCAAGTTGGACATCACCTCCCACAACGAGGACTACACCATCGTGGAACAGTACG<br>AACGCGCCGAGGGCCGCCACTCCACCGGCGGCATGGACGAGCTGTACAAGTAA	18
gfp	ATGCGTAAAGGCGAAGAGCTGTTCACTGGTGTCGTCCCTATTCTGGTGGAACTGGATGGTGATGTCAACGGTCATAAGTTTTCCGTGCGTGGCG<br>AGGGTGAAGGTGACGCAACTAATGGTAAACTGACGCTGAAGTTCATCTGTACTACTGGTAAACTGCCGGTACCTTGGCCGACTCTGGTAACGAC<br>GCTGACTTATGGTGTTCAGTGCTTTGCTCGTTATCCGGACCATATGAAGCAGCATGACTTCTTCAAGTCCGCCATGCCGGAAGGCTATGTGCAG<br>GAACGCACGATTTCCTTTAAGGATGACGGCACGTACAAAACGCGTGCGGAAGTGAAATTTGAAGGCGATACCCTGGTAAACCGCATTGAGCTGA<br>AAGGCATTGACTTTAAAGAAGACGGCAATATCCTGGGCCATAAGCTGGAATACAATTTTAACAGCCACAATGTTTACATCACCGCCGATAAACA<br>AAAAAATGGCATTAAAGCGAATTTTAAAATTCGCCACAACGTGGAGGATGGCAGCGTGCAGCTGGCTGATCACTACCAGCAAAACACTCCAATC<br>GGTGATGGTCCTGTTCTGCTGCCAGACAATCACTATCTGAGCACGCAAAGCGTTCTGTCTAAAGATCCGAACGAGAAACGCGATCATATGGTTC<br>TGCTGGAGTTCGTAACCGCAGCGGGCATCACGCATGGTATGGATGAACTGTACAAACATCATCATCATCATCATTGATAA	19
hok/sok	AACAAACTCCGGGAGGCAGCGTGATGCGGCAACAATCACACGGATTTCCCGTGAACGGTCTGAATGAGCGGATTATTTTCAGGGAAAGTGAGTG<br>TGGTCAGCGTGCAGGTATATGGGCTATGATGTGCCCGGCGCTTGAGGCTTTCTGCCTCATGACGTGAAGGTGGTTTGTTGCCGTGTTGTGTGGC<br>AGAAAGAAGATAGCCCCGTAGTAAGTTAATTTTCATTAACCACCACGAGGCATCCCTATGTCTAGTCCACATCAGGATAGCCTCTTACCGCGCT<br>TTGCGCAAGGAGAAGAAGGCCATGAAACTACCACGAAGTTCCCTTGTCTGGTGTGTGTTGATCGTGTGTCTCACACTGTTGATATTCACTTATC<br>TGACACGAAAATCGCTGTGCGAGATTCGTTACAGAGACGGACACAGGGAGGTGGCGGCTTTCATGGCTTACGAATCCGGTAAGTAGCAACCTGG<br>AGGCGGGCGCAGGCCCGCCTTTTCAGGACTGATGCTGGTCTGACTACTGAAGCGCCTTTATAAAGGGGCTGCTGGTTCGCCGGTAGCCCCTTTC<br>TCCTTGCTGATGTTGT	20
**Repeats**		
PP7X20	TAAGGTACCTAATTGCCTAGAAAGGAGCAGACGATATGGCGTCGCTCCCTGCAGGTCGACTCTAGAAACCAGCAGAGCATATGGGCTCGCTGGC<br>TGCAGTATTCCCGGGTTCATTAGATCCTAAGGTACCTAATTGCCTAGAAAGGAGCAGACGATATGGCGTCGCTCCCTGCAGGTCGACTCTAGAA<br>ACCAGCAGAGCATATGGGCTCGCTGGCTGCAGTATTCCCGGGTTCATTAGATCCTAAGGTACCTAATTGCCTAGAAAGGAGCAGACGATATGGC<br>GTCGCTCCCTGCAGGTCGACTCTAGAAACCAGCAGAGCATATGGGCTCGCTGGCTGCAGTATTCCCGGGTTCATTAGATCCTAAGGTACCTAAT<br>TGCCTAGAAAGGAGCAGACGATATGGCGTCGCTCCCTGCAGGTCGACTCTAGAAACCAGCAGAGCATATGGGCTCGCTGGCTGCAGTATTCCCG<br>GGTTCATTAGATCCTAAGGTACCTAATTGCCTAGAAAGGAGCAGACGATATGGCGTCGCTCCCTGCAGGTCGACTCTAGAAACCAGCAGAGCAT<br>ATGGGCTCGCTGGCTGCAGTATTCCCGGGTTCATTAGATCCTAAGGTACCTAATTGCCTAGAAAGGAGCAGACGATATGGCGTCGCTCCCTGCA<br>GGTCGACTCTAGAAACCAGCAGAGCATATGGGCTCGCTGGCTGCAGTATTCCCGGGTTCATTAGATCCTAAGGTACCTAATTGCCTAGAAAGGA<br>GCAGACGATATGGCGTCGCTCCCTGCAGGTCGACTCTAGAAACCAGCAGAGCATATGGGCTCGCTGGCTGCAGTATTCCCGGGTTCATTAGATC<br>CTAAGGTACCTAATTGCCTAGAAAGGAGCAGACGATATGGCGTCGCTCCCTGCAGGTCGACTCTAGAAACCAGCAGAGCATATGGGCTCGCTGG<br>CTGCAGTATTCCCGGGTTCATTAGATCCTAAGGTACCTAATTGCCTAGAAAGGAGCAGACGATATGGCGTCGCTCCCTGCAGGTCGACTCTAGA<br>AACCAGCAGAGCATATGGGCTCGCTGGCTGCAGTATTCCCGGGTTCATTAGATCCTAAGGTACCTAATTGCCTAGAAAGGAGCAGACGATATGG<br>CGTCGCTCCCTGCAGGTCGACTCTAGAAACCAGCAGAGCATATGGGCTCGCTGGCTGCAGTATTCCCGGGTTCATT	16
PhlFX14	TACATGATACGAAACGTACCGTATCGTTAAGGTGCATGATACGAAACGTACCGTATCGTTAAGGTTGTATGATACGAAACGTACCGTATCGTTA<br>AGGTGCATGATACGAAACGTACCGTATCGTTAAGGTCTGATGATACGAAACGTACCGTATCGTTAAGGTAAATGATACGAAACGTACCGTATCG<br>TTAAGGTGCATGATACGAAACGTACCGTATCGTTAAGGTCTGATGATACGAAACGTACCGTATCGTTAAGGTAAATGATACGAAACGTACCGTA<br>TCGTTAAGGTATGATCTAACTACTAGAGTACATGATACGAAACGTACCGTATCGTTAAGGTCATGATACGAAACGTACCGTATCGTTAAGGTTG<br>TATGATACGAAACGTACCGTATCGTTAAGGTGCATGATACGAAACGTACCGTATCGTTAAGGTCTGATGATACGAAACGTACCGTATCGTTAAG<br>GTATGATCTAAC	This study

Table S4: FISH probes used in this work

Target	Probe #	Sequence	Dye
PP7	1	cctttctaggcaattaggta	TAMRA
PP7	2	gagcgacgccatatcgtctg	TAMRA
PP7	3	atatgctctgctggtttcta	TAMRA
PP7	4	aatgaacccgggaatactgc	TAMRA
PP7	5	gcaattaggtaccttaggat	TAMRA
PP7	6	catatcgtctgctcctttct	TAMRA

References:

1. Paulsson J, Ehrenberg M. Noise in a minimal regulatory network: plasmid copy number control. *Q Rev Biophys* **34**, 1-59 (2001).

2. Young JW, *et al.* Measuring single-cell gene expression dynamics in bacteria using fluorescence time-lapse microscopy. *Nat Protoc* **7**, 80-88 (2011).

3. Skinner SO, Sepulveda LA, Xu H, Golding I. Measuring mRNA copy number in individual Escherichia coli cells using single-molecule fluorescent in situ hybridization. *Nat Protoc* **8**, 1100-1113 (2013).

4. Segall-Shapiro TH, Sontag ED, Voigt CA. Engineered promoters enable constant gene expression at any copy number in bacteria. *Nature biotechnology*, (2018).

5. Qi Lei S, *et al.* Repurposing CRISPR as an RNA-Guided Platform for Sequence-Specific Control of Gene Expression. *Cell* **152**, 1173-1183.

6. Meyer AJ, Segall-Shapiro TH, Glassey E, Zhang J, Voigt CA. Escherichia coli “Marionette” strains with 12 highly optimized small-molecule sensors. *Nature Chemical Biology*, (2018).

7. Munsky B, Neuert G, van Oudenaarden A. Using Gene Expression Noise to Understand Gene Regulation. *Science* **336**, 183-187 (2012).

8. Munsky B, Khammash M. The finite state projection algorithm for the solution of the chemical master equation. *J Chem Phys* **124**, 044104 (2006).

9. Shin J, Zhang S, Der BS, Nielsen AA, Voigt CA. Programming Escherichia coli to function as a digital display. *Molecular Systems Biology* **16**, e9401 (2020).

10. Schafer DA, Gelles J, Sheetz MP, Landick R. Transcription by single molecules of RNA polymerase observed by light microscopy. *Nature* **352**, 444-448 (1991).

11. Kim S, Beltran B, Irnov I, Jacobs-Wagner C. Long-Distance Cooperative and Antagonistic RNA Polymerase Dynamics via DNA Supercoiling. *Cell* **179**, 106-119 e116 (2019).

12. Deuschle U, Kammerer W, Gentz R, Bujard H. Promoters of Escherichia coli: a hierarchy of in vivo strength indicates alternate structures. *Embo J* **5**, 2987-2994 (1986).

13. Nielsen AA, *et al.* Genetic circuit design automation. *Science* **352**, aac7341 (2016).

14. Chen Y-J, *et al.* Characterization of 582 natural and synthetic terminators and quantification of their design

constraints. *Nat Meth* **10**, 659-664 (2013).

15. Lou C, Stanton B, Chen YJ, Munsky B, Voigt CA. Ribozyme-based insulator parts buffer synthetic circuits from genetic context. *Nat Biotechnol* **30**, 1137-1142 (2012).

16. Larson DR, Zenklusen D, Wu B, Chao JA, Singer RH. Real-time observation of transcription initiation and elongation on an endogenous yeast gene. *Science* **332**, 475-478 (2011).

17. Chen Y, Kim JK, Hirning AJ, Josic K, Bennett MR. SYNTHETIC BIOLOGY. Emergent genetic oscillations in a synthetic microbial consortium. *Science* **349**, 986-989 (2015).

18. Shaner NC, Campbell RE, Steinbach PA, Giepmans BN, Palmer AE, Tsien RY. Improved monomeric red, orange and yellow fluorescent proteins derived from Discosoma sp. red fluorescent protein. *Nat Biotechnol* **22**, 1567-1572 (2004).

19. Pedelacq JD, Cabantous S, Tran T, Terwilliger TC, Waldo GS. Engineering and characterization of a superfolder green fluorescent protein. *Nat Biotechnol* **24**, 79-88 (2006).

20. Chowdhury S, Castro S, Coker C, Hinchliffe TE, Arpaia N, Danino T. Programmable bacteria induce durable tumor regression and systemic antitumor immunity. *Nat Med* **25**, 1057-1063 (2019).
