# Supplementary material for: Single-cell measurement of plasmid copy number and promoter activity
Source: Nat Commun. 2021 Mar 5;12:1475. doi: 10.1038/s41467-021-21734-y (PMC7935883; doi:10.1038/s41467-021-21734-y)
Supplement: Supplementary file 3 — Reporting Summary [file 41467_2021_21734_MOESM3_ESM.pdf]

nature research

Corresponding author(s): Christopher A. Voigt

Last updated by author(s): Feb 5, 2021

Reporting Summary

Nature Research wishes to improve the reproducibility of the work that we publish. This form provides structure for consistency and transparency in reporting. For further information on Nature Research policies, see our Editorial Policies and the Editorial Policy Checklist.

Statistics

For all statistical analyses, confirm that the following items are present in the figure legend, table legend, main text, or Methods section.

n/a	Confirmed	
☐	☒	The exact sample size (*n*) for each experimental group/condition, given as a discrete number and unit of measurement
☐	☒	A statement on whether measurements were taken from distinct samples or whether the same sample was measured repeatedly
☐	☒	The statistical test(s) used AND whether they are one- or two-sided<br>*Only common tests should be described solely by name; describe more complex techniques in the Methods section.*
☒	☐	A description of all covariates tested
☒	☐	A description of any assumptions or corrections, such as tests of normality and adjustment for multiple comparisons
☐	☒	A full description of the statistical parameters including central tendency (e.g. means) or other basic estimates (e.g. regression coefficient) AND variation (e.g. standard deviation) or associated estimates of uncertainty (e.g. confidence intervals)
☐	☒	For null hypothesis testing, the test statistic (e.g. *F*, *t*, *r*) with confidence intervals, effect sizes, degrees of freedom and *P* value noted<br>*Give P values as exact values whenever suitable.*
☒	☐	For Bayesian analysis, information on the choice of priors and Markov chain Monte Carlo settings
☒	☐	For hierarchical and complex designs, identification of the appropriate level for tests and full reporting of outcomes
☒	☐	Estimates of effect sizes (e.g. Cohen's *d*, Pearson's *r*), indicating how they were calculated

Our web collection on statistics for biologists contains articles on many of the points above.

Software and code

Policy information about availability of computer code

Data collection	[flow] BD LSR Fortessa with BD FACSDiva 8.0; [microscope] Nikon Ti-E fluorescence microscopy with NIS-Elements 4.0 and Zeiss Axio Observer.Z1 microscopy with Zen Pro; [qPCR] Roche Lightcycler96 with SW 1.1; [FISH] Stellaris Probe Designer version 4.1.
Data analysis	[image processing] Schnitzcells software for cell segmentation. Spatzcells software and custom MATLAB script for spot quantification (https://github.com/VoigtLab/Promoter_Activity_Quantification); [flow cytometry] standard reporter level calculation using the FlowJo 7.6; [graphics] GraphPad Prism 7.

For manuscripts utilizing custom algorithms or software that are central to the research but not yet described in published literature, software must be made available to editors and reviewers. We strongly encourage code deposition in a community repository (e.g. GitHub). See the Nature Research guidelines for submitting code & software for further information.

Data

Policy information about availability of data

All manuscripts must include a data availability statement. This statement should provide the following information, where applicable:

- Accession codes, unique identifiers, or web links for publicly available datasets
- A list of figures that have associated raw data
- A description of any restrictions on data availability

Source data are provided with this paper. A list of figures with associated raw data: Fig 1, Fig 2, Fig 3, Fig S2, Fig S3, Fig S4, Fig S5, FIg S6, Fig S10, FIg S11, Fig S12, Fig S13, Fig S14, Fig S16 and Fig S17. Genetic part sequences are available in Supplementary Information. Key plasmids and strains are available from Addgene.

Field-specific reporting

Please select the one below that is the best fit for your research. If you are not sure, read the appropriate sections before making your selection.

☒ Life sciences ☐ Behavioural & social sciences ☐ Ecological, evolutionary & environmental sciences

For a reference copy of the document with all sections, see nature.com/documents/nr-reporting-summary-flat.pdf

Life sciences study design

All studies must disclose on these points even when the disclosure is negative.

Sample size	All main experiments were performed in triplicate on separate days. Statistical methods were not used to predetermine sample size. We found 3 biological replicates were sufficient for microscopy measurement and the results are highly reproducible across replicates.
Data exclusions	No data was excluded from analysis
Replication	All experimental claims have been tested three times on different days. All attempts at replication were successful.
Randomization	Cells were randomly selected for calculation of means without preconditioning.
Blinding	Blinding was not relevant for this study, since results from all constructs are analyzed.

Reporting for specific materials, systems and methods

We require information from authors about some types of materials, experimental systems and methods used in many studies. Here, indicate whether each material, system or method listed is relevant to your study. If you are not sure if a list item applies to your research, read the appropriate section before selecting a response.

Materials & experimental systems

n/a	Involved in the study	
☒	☐	Antibodies
☒	☐	Eukaryotic cell lines
☒	☐	Palaeontology and archaeology
☒	☐	Animals and other organisms
☒	☐	Human research participants
☒	☐	Clinical data
☒	☐	Dual use research of concern

Methods

n/a	Involved in the study	
☒	☐	ChIP-seq
☐	☒	Flow cytometry
☒	☐	MRI-based neuroimaging

Flow Cytometry

Plots

Confirm that:

☒ The axis labels state the marker and fluorochrome used (e.g. CD4-FITC).

☒ The axis scales are clearly visible. Include numbers along axes only for bottom left plot of group (a 'group' is an analysis of identical markers).

☒ All plots are contour plots with outliers or pseudocolor plots.

☒ A numerical value for number of cells or percentage (with statistics) is provided.

Methodology

Sample preparation	Bacterial cells were diluted into PBS containing Kanamycin to halt translation.
Instrument	BD LSR Fortessa
Software	Data was collected using FACS Diva software and FlowJo was used for data anaylsis
Cell population abundance	Typical samples contained 50,000 or more cells.

Gating strategy

The following gating strategy was used to separate the cells from debris: 3,500-200,000 SSC-A and 350-20,000 FSC-A

☒ Tick this box to confirm that a figure exemplifying the gating strategy is provided in the Supplementary Information.
